# Nervous and Muscular Adverse Events after COVID-19 Vaccination: A Systematic Review and Meta-Analysis of Clinical Trials

**DOI:** 10.3390/vaccines9080939

**Published:** 2021-08-23

**Authors:** Jiaxin Chen, Yuangui Cai, Yicong Chen, Anthony P. Williams, Yifang Gao, Jinsheng Zeng

**Affiliations:** 1 Department of Neurology, The First Affiliated Hospital, Guangdong Provincial Key Laboratory of Diagnosis and Treatment of Major Neurological Diseases, Sun Yat-sen University, Guangzhou 510080, China; chenjx239@mail2.sysu.edu.cn (J.C.); caiyg3@mail2.sysu.edu.cn (Y.C.); chenyc37@mail.sysu.edu.cn (Y.C.); 2 Department of Immunology, University Hospital Southampton NHS Foundation Trust, Southampton SO16 6YD, UK; apw3@soton.ac.uk; 3 Academic Unit of Cancer Sciences, Faculty of Medicine, University of Southampton and Southampton NIHR Experimental Cancer Medicine Centre, Southampton SO16 6YD, UK; 4 Organ Transplantation Center, The First Affiliated Hospital, Sun Yat-sen University, Guangzhou 510080, China

**Keywords:** COVID-19, vaccine, adverse events, nervous system, muscular system

## Abstract

**Background:** Nervous and muscular adverse events (NMAEs) have garnered considerable attention after the vaccination against coronavirus disease (COVID-19). However, the incidences of NMAEs remain unclear. We aimed to calculate the pooled event rate of NMAEs after COVID-19 vaccination. **Methods:** A systematic review and meta-analysis of clinical trials on the incidences of NMAEs after COVID-19 vaccination was conducted. The PubMed, Medline, Embase, Cochrane Library, and Chinese National Knowledge Infrastructure databases were searched from inception to 2 June 2021. Two independent reviewers selected the study and extracted the data. Categorical variables were analyzed using Pearson’s chi-square test. The pooled odds ratio (OR) with the corresponding 95% confidence intervals (CIs) were estimated and generated with random or fixed effects models. The protocol of the present study was registered on PROSPERO (CRD42021240450). **Results:** In 15 phase 1/2 trials, NMAEs occurred in 29.2% vs. 21.6% (*p* < 0.001) vaccinated participants and controls. Headache and myalgia accounted for 98.2% and 97.7%, and their incidences were 16.4% vs. 13.9% (OR = 1.97, 95% CI = 1.28–3.06, *p* = 0.002) and 16.0% vs. 7.9% (OR = 3.31, 95% CI = 2.05–5.35, *p* < 0.001) in the vaccine and control groups, respectively. Headache and myalgia were more frequent in the newly licensed vaccines (OR = 1.97, 95% CI = 1.28–3.06, *p* = 0.02 and OR = 3.31, 95% CI = 2.05–5.35, *p* < 0.001) and younger adults (OR = 1.40, 95% CI = 1.12–1.75, *p =* 0.003 and OR = 1.54, 95% CI = 1.20–1.96, *p* < 0.001). In four open-label trials, the incidences of headache, myalgia, and unsolicited NMAEs were 38.7%, 27.4%, and 1.5%. Following vaccination in phase 3 trials, headache and myalgia were still common with a rate of 29.5% and 19.2%, although the unsolicited NMAEs with incidence rates of ≤ 0.7% were not different from the control group in each study. **Conclusions:** Following the vaccination, NMAEs are common of which headache and myalgia comprised a considerable measure, although life-threatening unsolicited events are rare. NMAEs should be continuously monitored during the ongoing global COVID-19 vaccination program.

## 1. Introduction

As of 4 August 2021, over 199 million confirmed cases of coronavirus disease (COVID-19) have been reported, of which more than 4.2 million have resulted in death [1]. As the virus pandemic continues, mutations occur and resist the vaccine based on the prototype isolate. Despite vaccination, breakthrough infections have been observed [2]. A safe and effective vaccine against COVID-19 is a constructive medical strategy to protect susceptible populations, slow the spread, and restore normal social order. The willingness of the public to receive the COVID-19 vaccine has been surveyed and over 50% of participants either had a neutral attitude or questioned the safety of the vaccines [3,4,5]. With vaccine development gaining momentum, adverse events of vaccinations have attracted considerable attention. The commonly reported nervous and muscular adverse events (NMAEs), including headache and myalgia, and occasional cases of Bell’s palsy, myelitis, and cerebral venous thrombosis (CVT) after vaccination have caused some skepticism and panic, even suspension of vaccination [6,7,8,9,10]. Moreover, according to the European Database of suspected adverse drug reaction reports, NMAEs ranked in top and resulted in relatively high mortality in four experimental vaccines [11]. However, the incidences and state of NMAEs remain unclear. Therefore, NMAEs after vaccination must be summarized and analyzed to reduce panic and expand the acceptance and coverage of vaccines, especially since the threatening delta mutations of the virus have been observed in some countries [12,13]. Herein, we present a systematic review and meta-analysis of the clinical trials on the COVID-19 vaccine to evaluate the incidence of NMAEs after vaccination.

## 2. Materials and Methods

The guidelines of the Preferred Reporting Items for Systematic Reviews and Meta-Analysis guidelines were followed. The review was registered with PROSPERO (CRD42021240450) and reported according to PRISMA guidelines. The study protocol is available online.

### 2.1. Searches Strategy

A systematic search of the PubMed, Medline, Embase, Cochrane Library, and Chinese National Knowledge Infrastructure databases was performed from the inception to 2 June 2021 using the Medical Subject Headings and the terms “COVID-19”; “vaccine”; “clinical trials”. There were no language restrictions. Additionally, the official websites of the vaccine developers were also searched.

### 2.2. Study Selection Criteria

The title and abstract of the identified publications were screened, and any potentially eligible articles were retrieved for a full-text review. The inclusion criteria were (1) in terms of population, individuals enrolled in the clinical trials of COVID-19 vaccine (including the vaccine and control groups); (2) study designs were controlled clinical trials; (3) outcomes were the safety of COVID-19 vaccines and the incidence of NMAEs after vaccination. The exclusion criteria were (1) reviews, systematic reviews, meta-analysis, editorial, news, conference proceedings, protocols, articles on diagnoses or drug treatments and (2) articles focused on immunogenicity or efficacy without complete data on adverse events. Two reviewers (J.C. and Y. Cai) independently performed the study selection, and disagreements were resolved through discussion or according to the judgment of a third reviewer (Y. Chen). In the case of multiple reports from the same data set, the most recent or comprehensive report was selected.

### 2.3. Data Extraction

For each included clinical trial, data on the study and patient characteristics were extracted independently and in duplicate (J.C. and Y. Cai) using a standardized data extraction sheet; afterward, the results were cross-checked. Discrepancies were resolved by consensus or with the judgment of a third reviewer (Y. Chen). The extracted study and patient characteristics included the publication dates, countries, vaccination platforms, population, participants’ age, sample sizes, vaccines doses, placebo/control, and research stages. The inactivated vaccine was classified as traditional vaccine, recombinant protein vaccine, replication-incompetent vectors vaccine, and mRNA vaccine as newly licensed vaccines [14]. According to the regulation on adverse events, NMAEs would be divided into the solicited and unsolicited ones [15,16]. The solicited NMAEs were listed in the trial protocol and documented by diary card to ensure they were informed, while the unsolicited NMAEs were unforeseeable and reported on participants’ own initiative. The primary outcome was the incidences of NMAEs after COVID-19 vaccination, including the solicited and unsolicited ones. Except for annotation, the numbers of NMAEs were person-time, and a person who experienced vaccination twice would be recorded as 2 in this study.

### 2.4. Quality Assessment

The study quality assessment was performed according to the validated scale for randomized controlled trials recommended by Cochrane [17]. For each clinical trial, two of the reviewers (J.C. and Y. Cai) assigned independent scores of high, low, or unclear to each of the following domains: sequence generation, allocation concealment, blinding of participants, blinding of outcome assessments, incomplete outcome data, selective reporting, and other biases. Discrepancies in the quality assessments were resolved by consensus or by a third reviewer (Y. Chen). We included all the eligible clinical trials, regardless of their assessed quality.

### 2.5. Statistical Analysis

NMAEs after COVID-19 vaccination were analyzed using Pearson’s chi-square test for categorical variables with SPSS 23.0 (SPSS Inc., Chicago, IL, USA). In the meta-analysis, for each of the included studies, the differences in the frequencies of NMAEs with vaccine versus control or baseline were pooled, stratified across the studies, and analyzed using random-effects or fixed-effects models with inverse variance weighting. Random-effects models were used when the *I*^2^ statistics were used to estimate the proportion of variation attributable to between-study heterogeneity of >50% or *p* < 0.1. Fixed-effects models were used when the *I*^2^ was <50% or *p* > 0.5. The pooled effects on NMAEs were presented as odds ratios (ORs) with corresponding 95% confidence intervals (CIs). The statistical analyses were performed using Review Manager (version 5.2; Copenhagen: The Nordic Cochrane Centre, The Cochrane Collaboration, 2012). Publication bias was visualized by funnel plots and measured by the Begg–Mazumdar rank correlation and the Egger bias test conducted using STATA (version 11.2, StataCorp, College Station, TX 77845, USA).

## 3. Results

**Figure 1:**
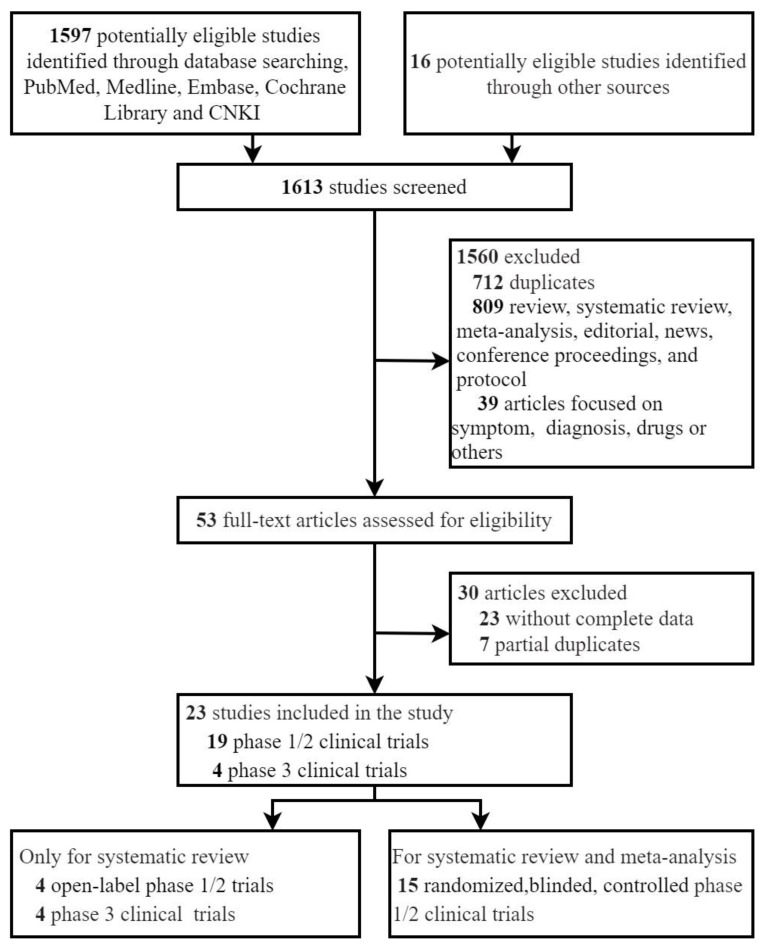
PRISMA flowchart describing systematic literature search and study selection. Data of some vaccines published in the developer’s official websites or pre-print were duplicated with the published articles. The included phase 3 clinical trials partly contained data of phase 1 and/or 2. CNKI = Chinese National Knowledge Infrastructure databases.

We identified 1613 studies from the databases and manual searches (Figure 1). After the exclusion of 712 duplicates, 901 articles were reviewed based on their titles and abstracts, of which 848 articles were excluded based on the article type (reviews, systematic reviews, editorials, news, protocol and conference proceeding), and topic (symptom, diagnosis, drug, or others). A total of 53 full-text articles were assessed for eligibility, of which 30 were excluded. In total, 23 studies did not report the complete and clear data of NMAEs, and the other 7 studies were the pre-prints or the data subsets to some newer and more comprehensive studies. Finally, 23 articles met the inclusion criteria and were included in the systematic review and meta-analysis [18,19,20,21,22,23,24,25,26,27,28,29,30,31,32,33,34,35,36,37,38,39,40]. The baseline characteristics of the 23 included studies are summarized in Table 1. Overall, 15 randomized, blinded, controlled phase 1/2 clinical trials revealed a low risk of bias (Figure S1) and enrolled in the systematic review and meta-analysis [18,19,20,21,22,23,24,25,26,27,28,29,30,31,32]. Four open-label, without placebo-controlled phase 1/2 trials and four phase 3 clinical trials were only included in the systematic review [33,34,35,36,37,38,39,40]. The solicited and unsolicited NMAEs were clearly illustrated and can be extracted from all included studies.

All reported NMAEs occurred within the period under the safety observation; most were 7 days, and others were 14 or 28 days. The incidence of NMAEs was 29.2% in the vaccine group and 21.6% in the control (*p* < 0.001) in a total of 15 randomized, blinded, controlled clinical trials (Table S1). The *I*^2^ was 92% in the total NMAEs of 15 studies, and meta-analysis could not be performed.

**Figure 2:**
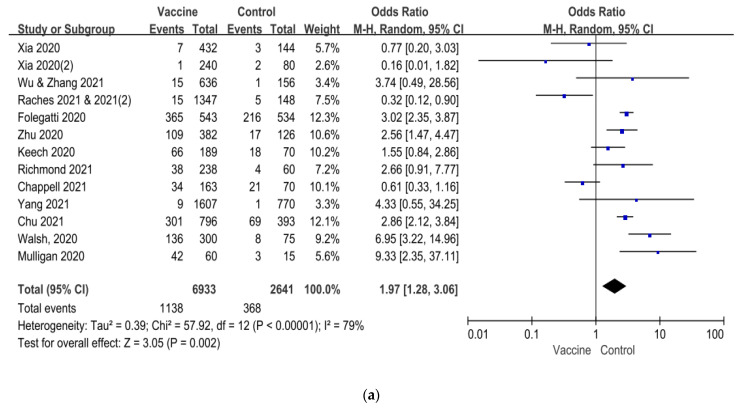

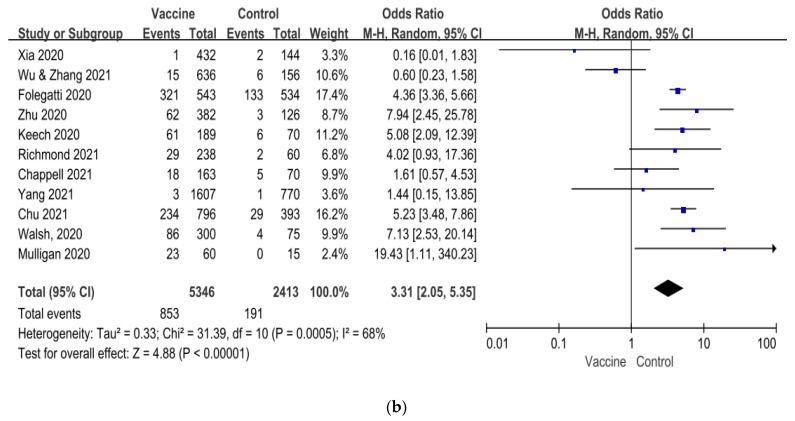
Forest plot analysis of the incidence of headache (**a**) and myalgia (**b**) on the vaccine and control groups. An odd ratio of >1 is indicative of a higher incidence of headache and myalgia in the vaccine group, compared to the control group. A Mante–Haenszel variance random-effects model (M–H, random) was employed to estimate effects between vaccine and control groups. I^2^ statistic, 95% confidence interval (CI), and point estimates are displayed and diamond is presented the pooled effect. Forest plot analysis of the incidence of headache (**a**) and myalgia (**b**) on the vaccine and control groups. An odd ratio of >1 is indicative of a higher incidence of headache and myalgia in the vaccine group, compared to the control group. A Mante–Haenszel variance random-effects model (M–H, random) was employed to estimate effects between vaccine and control groups. I^2^ statistic, 95% confidence interval (CI), and point estimates are displayed and diamond is presented the pooled effect.

**Figure 3:**
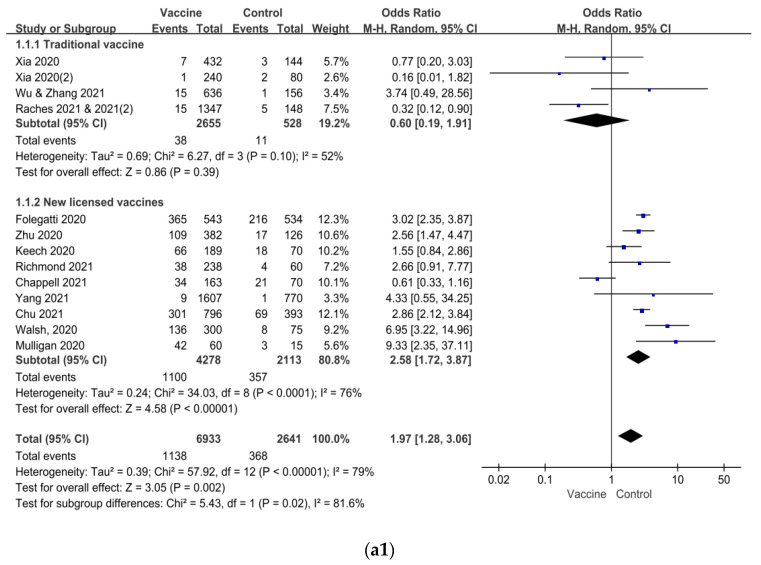

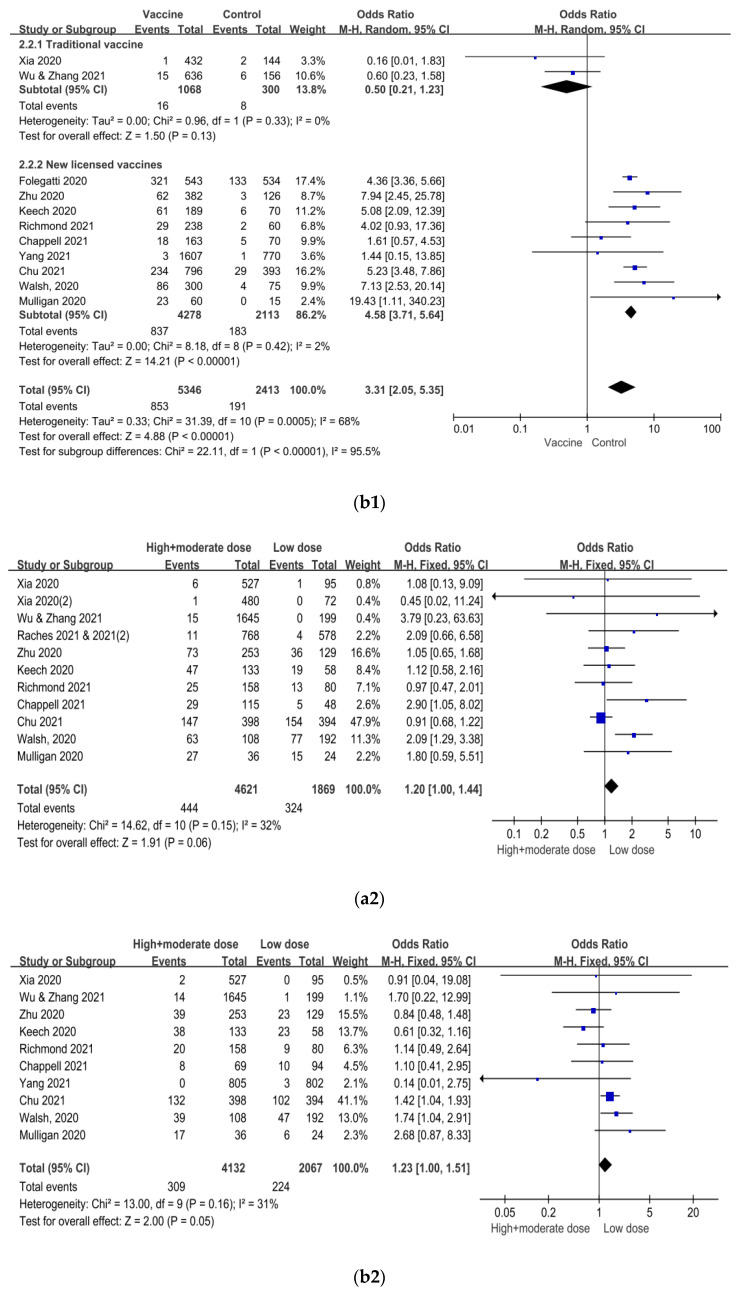

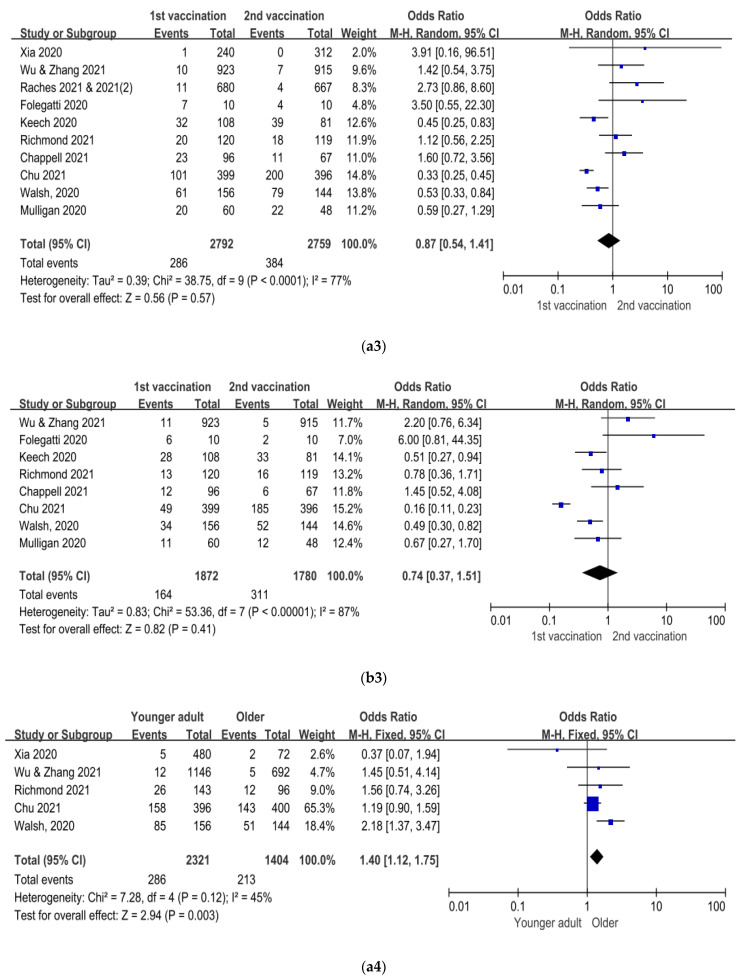

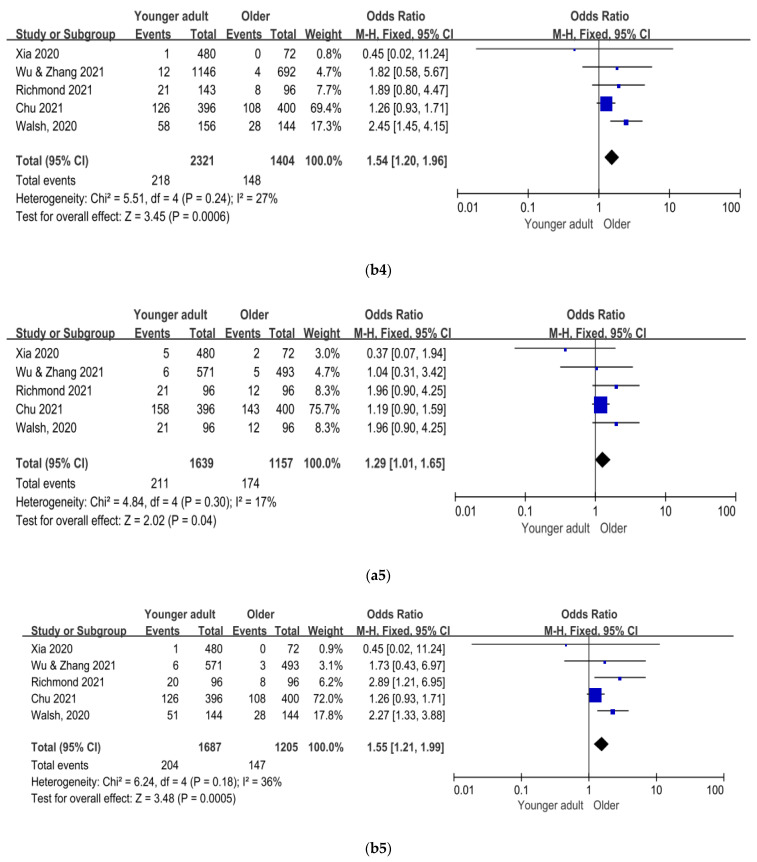
Subgroup analysis of headache and myalgia. Vaccine platform on headache (**a1**) and myalgia (**b1**); vaccine dose on headache (**a2**) and myalgia (**b2**); the 1st and 2nd vaccination on headache (**a3**) and myalgia (**b3**); age of participant on headache (**a4**) and myalgia (**b4**); age of participant (adjusted) on headache (**a5**) and myalgia (**b5**). Traditional vaccine is inactivated vaccine, and the newly licensed vaccines include replication-incompetent vectors vaccine, recombinant protein vaccine, and mRNA vaccine. An odd ratio of >1 is indicative of a higher incidence of headache and myalgia in the vaccine group, compared to the control group. A Mantel–Haenszel variance random-effects model (M–H, random) was employed to estimate effects between vaccine and control groups. I^2^ statistic, 95% confidence interval (CI), and point estimates are displayed and diamond is presented the pooled effect.

However, both headache and myalgia accounted for 98.2% and 97.7%, while the remaining others, including dizziness, drowsiness, hypoesthesia, etc., only accounted for 1.8% and 2.3% in the vaccine and control groups, respectively. As the solicited adverse reaction of the safety set, headache and myalgia had more detailed information recorded and low published bias for meta-analysis, although the *I*^2^ was 79% and 68%, respectively (Figure S2). The incidences of headache and myalgia were 16.4% vs. 13.9% (OR = 1.97, 95% CI = 1.28–3.06, *p* = 0.002) and 16.0% vs. 7.9% (OR = 3.31, 95% CI = 2.05–5.35, *p* < 0.001) in vaccine and control groups, respectively (Figure 2). In the subgroup analysis (Figure 3), the newly licensed vaccines had more headache and myalgia, compared with the control groups (OR = 2.58, 95% CI = 1.72–3.87, *p* < 0.001 and OR = 4.58, 95%CI = 3.71–5.64, *p* < 0.001, respectively), while the inactivated vaccines had no differences. Differences in the incidences of headache and myalgia were significant neither between the first and second vaccination doses nor between the high, moderate, and low doses (all *p* > 0.05). However, headache and myalgia were more frequent in younger adults than in the older participants (OR = 1.40, 95% CI = 1.12–1.75, *p* *=* 0.003 and OR = 1.54, 95% CI = 1.20–1.96, *p* < 0.001, respectively). After excluding the extra high-dose group, headache and myalgia were significantly different between the younger and older (OR = 1.29, 95% CI = 1.01–1.65, *p* = 0.04 and OR = 1.55, 95% CI = 1.21–1.99, *p <* 0.001, respectively). In a study by Folegatti [24], headache and myalgia were not significantly different with or without prophylactic paracetamol, in either the vaccine or the control groups (Table S2).

In four open-label, without placebo-controlled clinical trials, the incidences of headache, myalgia, and other NMAEs were 38.7%, 27.4%, and 1.5%, respectively (Table S3) [33,34,35,36].

In the four phase 3 clinical trials, the person-times of NMAEs were reported, while the vaccination times in participants were disarranged [37,38,39,40]. The incidences of headache and myalgia were 29.5% vs. 21.0% (*p* < 0.001) and 19.2% vs. 8.4% (*p* < 0.001) in the vaccine and control groups, respectively (Table S4). In the study by Baden [40], headache and myalgia were more common after the second vaccination and younger adults than those after the first vaccination and in older groups (all *p <* 0.05, Table S5). In addition, this study showed the duration of solicited adverse reactions after vaccination. In the vaccine group, headache and myalgia lasted (2.1 ± 2.2) days and (2.3 ± 3.2) days after the first vaccination, and (2.3 ± 2.9) days and (2.1 ± 3.1) days after the second dosage, respectively.

The highest incidence of unsolicited NMAEs after COVID-19 vaccination was 0.7%, and all of them were not significantly different from the controls in phase 3 clinical trials (all *p >* 0.05, Table 2). In the inactivated vaccine, cranial nerve lesions mainly manifested as dysphagia, accounted for 97.3% of all the unsolicited NMAEs [37]. In one study of the replication-incompetent vectors vaccines, sensory disorders were 68.2% of the unsolicited NMAEs. In two cases of transverse myelitis, one was considered possibly related to the vaccine, the other was considered potentially related at first, then determined to be unlikely related to the vaccine [38]. In the other study of replication-incompetent vectors vaccines, the cranial nerve lesions including taste disorders, visual impairments, and noises in the ears, were reported after the second vaccination dose [39]. In the study of the mRNA vaccine, three of the four Bell’s palsy occurred in the vaccine group over 28 days after vaccination, which is not listed in Table 2 [40].

## 4. Discussion

Although the emergency use authorization for some COVID-19 vaccines has been approved by the government and World Health Organization, NMAEs after vaccination have yet been comprehensively discussed. In the present study, we found that NMAEs occurred in 29.2% of the vaccinated participants in phase 1/2 clinical trials, with headache and myalgia being the most common and more frequent in the newly licensed vaccines and younger adults. In each phase 3 clinical study, headache and myalgia were still common, although the unsolicited NMAEs with incidence rates of ≤0.7% were not significantly different from the controls. These results indicate that following the COVID-19 vaccination, NMAEs, especially headache and myalgia, were common, although the severe life-threatening ones were rare.

Subgroup analysis demonstrated that the incidences of headache and myalgia were more common in the newly licensed vaccines than those in the controls but not in the inactivated vaccine, which means that inactivated vaccine may have favorable safety and tolerability [41,42,43,44]. The previous studies suggest that RNA-based vaccines had higher side effects in reactogenicity. The adenovirus-vectored vaccines are associated with increased diarrhea and arthralgia. Inactivated vaccines had fewer side effects, which may be associated with the mechanism, the mature technology, the alum-adjuvanted, or other factors [42,45]. However, this has resulted from the limited data of phase 1/2 clinical trials and a high level of heterogeneity between the studies. The data from the ongoing phase 3 trials deserve expectation.

Generally, the dosage is closely related to adverse reactions. However, in the current study, the incidences of headache and myalgia had no differences between the high, moderate dose and low dose, or between the first and second vaccination doses. No significant correlation between the dose and adverse events occurrence was found, consistent with the previous literature about COVID-19 vaccines [43,46]. Different vaccines have varied doses. The number of active ingredients contained in each milliliter vaccine should be used as the basis for comparing doses between different vaccines but not the total volume including various adjuvant [44]. A lack of standardized dosage grading means cross-comparison of safety, and the dose is limited. Additionally, different trials had different periods for observation of the safety, most of which were 7 days, and others were 14 or 28 days. Therefore, the dose-related safety required more accurate vaccine dosage and restricted follow-up time.

Another related factor of safety that deserves attention is the age of the participant. Here, the included participants of COVID-19 vaccines were healthy adults above 18 years of age. However, the majority of clinical trials did not cover population > 60 years of age that is particularly at risk for illness and death from COVID-19 [47,48]. We found that the incidences of headache and myalgia were significantly higher in younger adults than in older. Previous studies observed that binding-antibody levels in those above 70 years of age were low after COVID-19 vaccination [31,49]. This may indicate that the immune responses in the older population are relatively lower, and age-related immunosenescence may account for the low incidence of adverse events [6,7,50]. Although we did not analyze the binding-antibody levels after vaccination, these results suggest that the older individuals vulnerable to COVID-19 are not at risk of available vaccination.

Since few studies have reported the detailed date, the onset and lasting time of headache and myalgia after vaccination cannot be concluded. Safety data on COVID-19 vaccines need to be disclosed further to implement the appropriate measures quickly.

The neurotropism of the coronavirus and its systematic or focal neurological complications, including headache, cranial nerve lesions (anosmia and dysgeusia), stroke, myelitis, etc., have been well recognized [51,52]. Additionally, the spike protein, the receptor-binding domain, and other structural proteins have become the antigenic target for COVID-19 vaccines, which have an essential role in inducing specific immune responses [14]. In the four phase 3 clinical trials, headache and myalgia still ranked in the top of NMAEs and could not be prevented by paracetamol, but most of them were relieved spontaneously without treatment. However, other NMAEs were low and had no statistical differences between the vaccine and control groups. Although 17 cases of cerebrovascular events were recorded, no definite CVT was reported. Recently, CVT following COVID-19 vaccination has been a topic of concern in two mRNA vaccines and one replication-incompetent vector vaccine [9,10,53,54]. The events occurred more in women under 60 years of age with headaches initially after vaccination, similar to the other typical CVT [9,10,53,54]. Therefore, severe and persistent headaches after vaccination could be the initial and unique symptom of CVT and should be considered and continuously monitored during the ongoing global COVID-19 vaccination program [10,55].

Several limitations are inherent to this study. As with all systematic reviews and meta-analyses, the potential for publication bias exists. Additionally, there was a high level of heterogeneity between the studies and a lack of more detailed information and control groups in some trials. Such unmeasured confounding variables may have influenced our results. The related data update quickly and have a possibility of omission. Moreover, the follow-up time of phase 1/2 clinical trials was not long enough, and their phase 3 trials are still ongoing. Despite these limitations, in this systemic review and meta-analysis, we calculated and analyzed the pooled event rate of NMAEs in the COVID-19 vaccines, providing a theoretical basis and recommendations for subsequent development and clinical applications.

## Figures and Tables

**Table 1:** Baseline characteristics of the included clinical trials of COVID-19 vaccines.

Author/ Year of Published	Vaccine Platform	Sample Size of Study	Age of Subjects (Year)	Dosage of Vaccine	Number of Vaccination	Control	Current Status of Clinical Trial
**Randomized, blinded, controlled phase 1/2 clinical trials**							
Xia 2020 [18]	Inactivated vaccine	Phase 1: 96, Phase 2: 448	18~80	2 µg, 4 µg, 8 µg	1 or 2	Saline containing aluminium hydroxide	Phase 3 ongoing
Xia 2020(2) [19]	Inactivated vaccine	Phase 1: 96, Phase2: 224	18~59	2.5 µg, 5 µg, 10 µg	2 or 3	Aluminum hydroxide (alum) adjuvant	Phase 3 ongoing
Wu 2021 [20] and Zhang 2021 [21]	Inactivated vaccine	18~59:744 ≥60: 422	18~59, ≥60	1.5 µg, 3 µg, 6 µg	2	Aluminium hydroxide diluent solution	Phase 3 ongoing
Raches 2021 [22] and Raches 2021(2) [23]	Inactivated vaccine	375	18~55	3 µg, 6 µg	2	Sterile solution and adjuvants	Phase 2 ongoing
Folegatti 2020 [24]	Replication-incompetent vectors vaccine	1077	18~55	5 × 10^10^ viral particles/mL	1 or 2	MenACWY vaccine	Suspended in some regions
Zhu 2020 [25]	Replication-incompetent vectors vaccine	508	≥18	1 × 10^11^ or 5 × 10^10^ viral particles/mL	1	Vaccine excipients	Phase 3 ongoing
Keech 2020 [26]	Recombinant protein vaccine	131	18~59	5 µg, 25 µg	1 or 2	Saline	Phase 3 in preparation
Richmond 2021 [27]	Recombinant protein vaccine	151	18~54, 55~75	3 µg, 9 µg, 30 µg	2	Saline	Phase 2 ongoing
Chappell 2021 [28]	Recombinant protein vaccine	120	18~55	5 µg, 15 µg, 45 µg	1 or 2	Saline	Phase 2 ongoing
Yang 2021 [29]	Recombinant protein vaccine	Phase 1: 50, Phase2: 900	18~59	25 µg, 50 µg	2 or 3	Aluminium hydroxide in buffer	Phase 3 ongoing
Chu 2021 [30]	mRNA vaccine	600	18~55, ≥55	50 µg, 100 µg	2	Saline	Phase 3 ongoing
Walsh 2020 [31]	mRNA vaccine	195	18~55, 65~85	10 µg, 20 µg, 30 µg, 100 µg	1 or 2	Saline	Phase 3 ongoing
Mulligan 2020 [32]	mRNA vaccine	45	18~55	10 µg, 30 µg, 100 µg	1 or 2	Saline	Phase 3 ongoing
**Open-label phase 1/2 clinical trials**							
Logunov 2020 [33]	Replication-incompetent vectors vaccine	76	18~60	Vac 0.5 mL, Vac-Lyo 1.0 mL	1 or 2	No, open-label	Phase 3 ongoing
Zhu 2020(2) [34]	Replication-incompetent vectors vaccine	108	18~60	5 × 10^11^ or 1 × 10^11^ or 5 × 10^10^ viral particles/mL	1	No, open-label	Phase 3 ongoing
Jackson 2020 [35] and Anderson 2020 [36]	mRNA vaccine	85	18~55, 56~70, ≥71	25 µg, 100 µg, 250 µg	2	No, open-label	Phase 3 ongoing
Phase 3 clinical trials *							
Kaabi 2021 [37]	Inactivated vaccine	40,411	≥18	5 µg, 4 µg	2	Alum adjuvant and saline	Ongoing
Voysey 2020 [38]	Replication-incompetent vectors vaccine	23,843	≥18	(3.5–6.5) × 10^10^ or 2.2 × 10^10^ viral particles/mL	2	MenACWY vaccine	Suspended in some regions
Logunov 2021 [39]	Replication-incompetent vectors vaccine	21,977	≥18	0.5 mL	2	Vaccine buffer	Ongoing
Baden 2020 [40]	mRNA vaccine	30,420	≥18	100 µg	2	Saline	Ongoing

*: All studies included the data of phase 3 clinical trials, partly contained data of phase 1/2. MenACWY vaccine = meningococcal group A, C, W, and Y conjugate vaccine.

**Table 2:** Other nervous and muscular adverse events after COVID-19 vaccination in phase 3 clinical trials.

	Inactivated Vaccine			Replication-Incompetent Vectors Vaccine						mRNA Vaccine	
	Kaabi 2021 [37]			Voysey 2020 [38]		Logunov 2021 [39]				Baden 2020 [40]	
	Vaccine		Control *n* = 13,453	Vaccine *n* = 12,021	Control *n* = 11,724	Vaccine		Control		Vaccine *n* = 15,185	Control *n* = 15,166
	WIV04 *n* = 13,464	HB02 *n* = 13,471				At Least One Dose *n* = 16,427	Two Dose *n* = 9258	At Least One Dose *n* = 5435	Two Dose *n* = 3038		
Systemic neurological symptoms *	0	1	0	9	10	1	NA	0	NA	14	19
Confusional state, drowsiness	NA	NA	NA	NA	NA	NA	1	NA	0	2	0
Seizure/tonic convulsion	NA	NA	NA	NA	NA	NA	NA	NA	NA	0	2
Cranial nerve lesions ^†^	51	58	62	6	10	NA	6	NA	1	NA	NA
Cerebrovascular events ^‡^	NA	NA	NA	2	4	3	NA	2	NA	5	1
Spinal cord events ^§^	0	2	0	4	2	0	NA	1	NA	NA	NA
Motor disorder ^¶^	NA	NA	NA	1	3	1	NA	0	NA	3	3
Sensory disorder ^||^	NA	NA	NA	60	63	NA	1	NA	1	2	0
Neuralgia, neuritis	NA	NA	NA	4	1	NA	NA	NA	NA	NA	NA
Muscle spasms/ facial spasm	NA	NA	NA	1	0	NA	NA	NA	NA	0	2
Autonomic nerve dysfunction **	NA	NA	NA	1	0	1	1	0	0	0	1
Total, No. (%)	51 (0.4)	61 (0.5)	62 (0.5)	88 (0.7)	93 (0.8)	6 (<0.1)	9 (<0.1)	3 (<0.1)	2 (<0.1)	26 (0.2)	28 (0.2)

* Included migraine, dizziness, vertigo, syncope, presyncope, muscle weakness, pathological changes in nervous system. ^†^ Included facial paralysis, Bell’s palsy, visual impairment, blindness transient, noise in ears, metallic taste, dysphagia. ^‡^ Included ischemic stroke, hemorrhagic stroke, cerebrovascular accident, embolic stroke, subarachnoid hemorrhage, transient ischemic attack, cerebral circulation failure, vertebrobasilar insufficiency, vascular encephalopathy. ^§^ Included myelitis, myelitis transverse, multiple sclerosis attacks, clinically isolated syndrome, acute disseminated encephalomyelitis. ^¶^ Included monoparesis, hemiparesis, gait disturbance, vestibular ataxia, fall. ^||^ Included paresthesia, sensory disturbance, sensory loss, hyperesthesia, hypoesthesia, dysesthesia. ** Included anal incontinence, disorder of autonomic nerve system. NA = not available. No. = number.

## Data Availability

The datasets used and analyzed during the current study are available from the corresponding author on reasonable request.

## Supplementary Materials

The following are available online at https://www.mdpi.com/article/10.3390/vaccines9080939/s1. Figure S1: Risk-of-bias graph and risk-of-bias summary of the included studies, Figure S2: Funnel diagram, Begg–Mazumdar rank correlation, and Egger’s bias test of publication bias of headache and myalgia, Table S1: Nervous and muscular adverse events in phase 1/2 randomized, blinded, and controlled clinical trial after COVID-19 vaccination, Table S2: The incidences of headache and myalgia with or without prophylactic paracetamol, Table S3: Nervous and muscular adverse events in phase 1/2 open-label clinical trial of COVID-19 vaccination, Table S4: Headache and myalgia in phase 3 clinical trial of COVID-19 vaccination, Table S5: Headache and myalgia subgroup in study of Baden 2020.
