# Supplementary material for: Nervous and Muscular Adverse Events after COVID-19 Vaccination: A Systematic Review and Meta-Analysis of Clinical Trials
Source: Vaccines (Basel). 2021 Aug 23;9(8):939. doi: 10.3390/vaccines9080939 (PMC8402736; doi:10.3390/vaccines9080939)
Supplement: Supplementary file 1 [file vaccines-09-00939-s001.zip › vaccines-1319900-supplementary.pdf]

Supplementary Materials

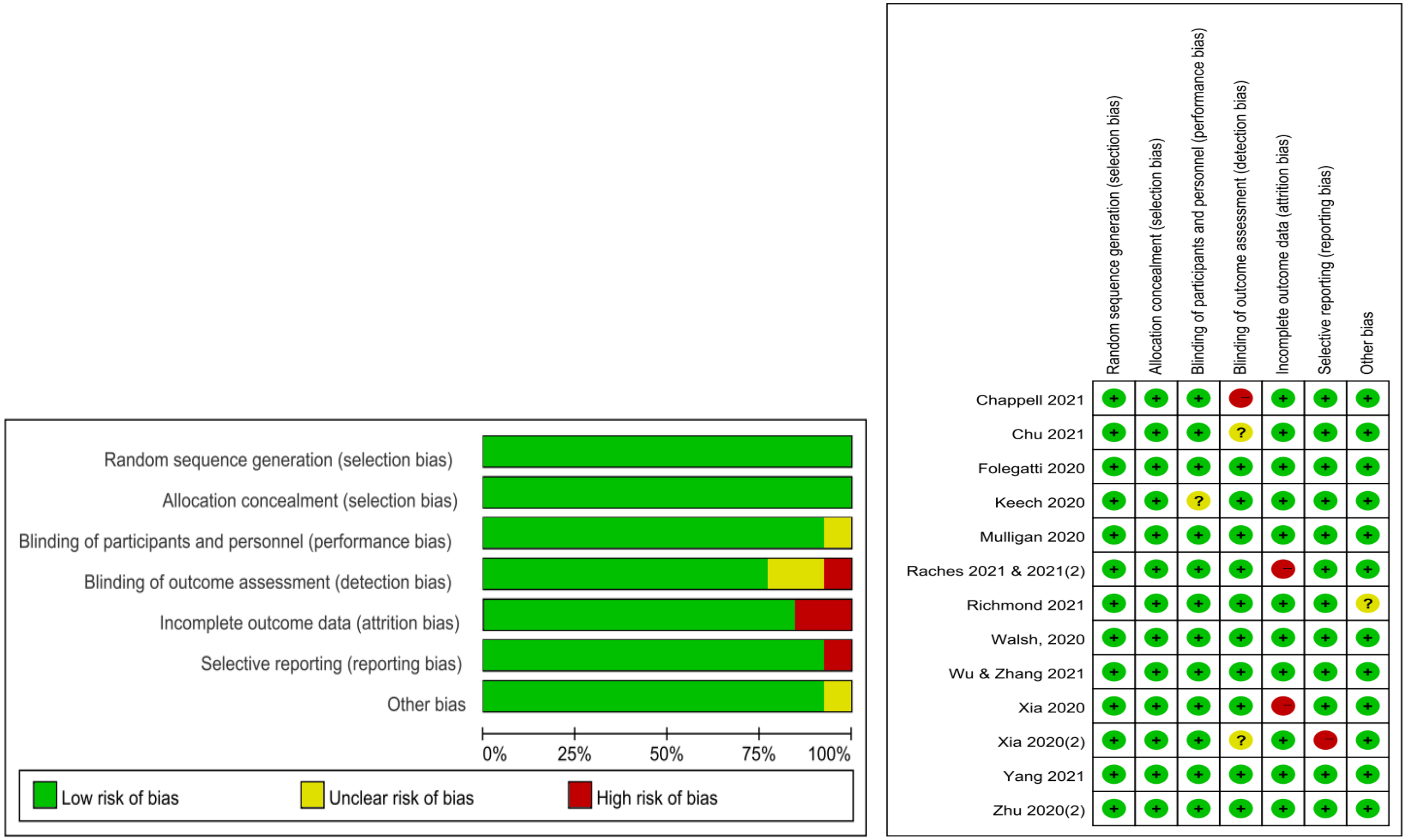

Figure S1. Risk of bias graph and risk of bias summary of the included studies.

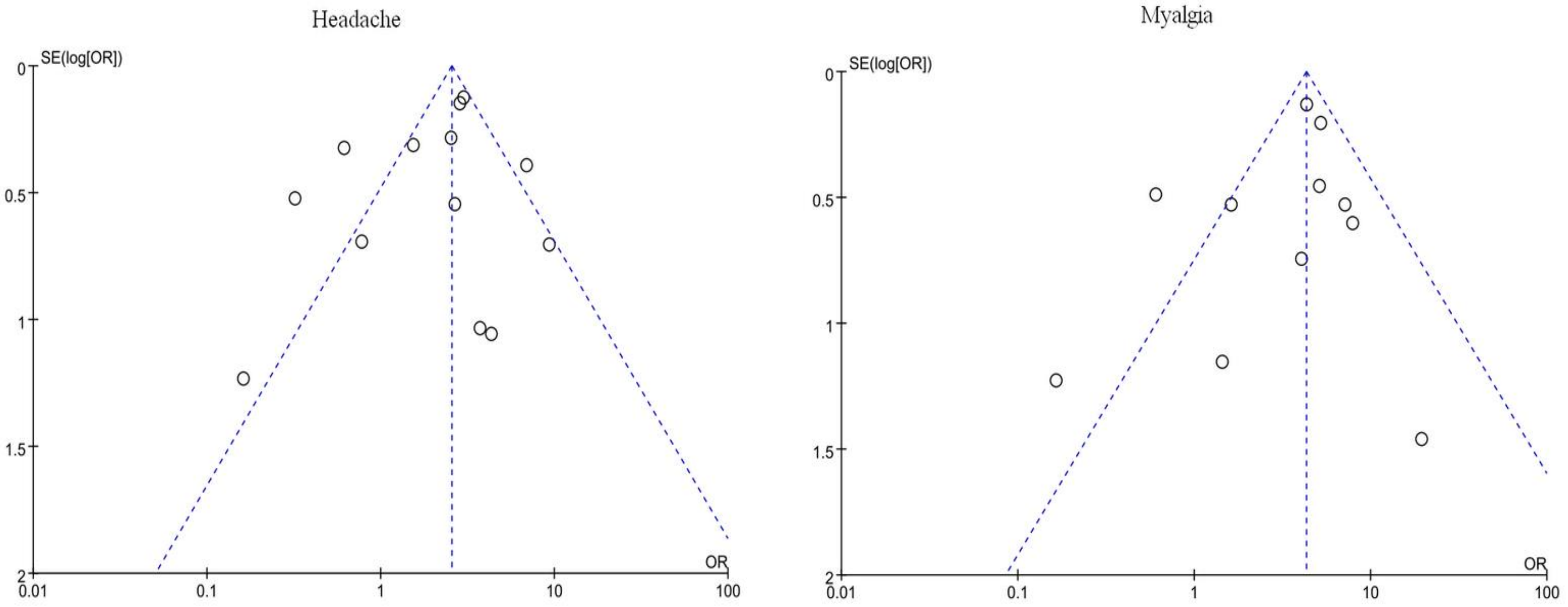

	Headache	Myalgia
Begg-Mazumdar rank correlation, Pr＞/z/	0.583	0.640
Egger's Test, P＞/t/	0.283	0.311

Figure S2. Funnel diagram, Begg-Mazumdar rank correlation and Egger's bias test of publication bias of headache and myalgia

Table S1. Nervous and muscular adverse events in phase 1/2 randomized, blinded, and controlled clinical trial after COVID-19 vaccination

Author/ year of published	Events	No. of events/total (%)	
		Vaccine	Control
Xia 2020 [1]	Headache	7/432 (1.6)	3/144 (2.1)
	Myalgia	1/432 (0.2)	2/144 (1.4)
	Drowsiness	1/432 (0.2)	0/144 (0)
	Dizziness	1/432 (0.2)	0/144 (0)
Xia 2020(2) [2]	Headache	1/240 (0.4)	2/80 (2.5)
Wu 2021 [3] and Zhang 2021 [4]	Headache	15/636 (2.4)	1/156 (0.6)
	Myalgia	15/636 (2.4)	6/156 (3.8)
	Hypoesthesia	2/636 (0.3)	2/156 (1.3)
	Dizziness	2/636 (0.3)	0/156 (0)
	Drowsiness	2/636 (0.3)	1/156 (0.6)
Raches 2021[5] and Raches 2021(2) [6]	Headache	15/1347 (1.1)	5/148 (3.4)
	Tremor	4/1347(0.3)	0/148 (0)
	Dizziness	1/1347 (0.1)	1/148 (0.7)
	Sleep disturbance	1/1347 (0.1)	0/148 (0)
	Giddiness	2/1347 (0.1)	0/148 (0)
	Vertigo	0/1347 (0)	0/148(0)
Folegatti 2020 [7]	Headache	365/543 (67.2)	216/534 (40.4)
	Myalgia	321/543 (59.1)	133/534 (24.9)
	Feeling abnormal	1/543 (0.2)	0/534 (0)
	Muscle spasms/ musculoskeletal stiffness	2/543 (0.4)	0/534 (0)
	Abnormal sleep-related event/ insomnia	1/543 (0.2)	1/534 (0.2)
	Dysgeusia	0/543 (0)	1/534 (0.2)
	Paraethesia	1/543 (0.2)	0/534 (0)
	Dizziness	2/543 (0.4)	0/534 (0)
Zhu 2020 [8]	Headache	109/382 (28.5)	17/126 (13.5)
	Myalgia	62/382 (16.2)	3/126 (2.4)
	Dizziness	5/382 (1.3)	1/126 (0.8)
	Hypersensitivity	0/382 (0)	2/126 (1.6)
	Syncope	2/382 (0.5)	0/126 (0)
	Hypoaesthesia	1/382 (0.3)	0/126 (0)
	Insomnia	1/382 (0.3)	0/126 (0)
	Somnolence	2/328 (0.5)	1/126 (0.8)
Keech 2020 [9]	Headache	66 /189 (30.7)	18/70 (25.7)
	Myalgia	61 /189 (28.4)	6 /70 (8.6)
Richmond 2021 [10]	Headache	38/238 (16.0)	4 /60 (6.7)
	Myalgia	29/238 (12.2)	2 /60 (3.3)
Chappell 2021 [11]	Headache	34/163 (20.9)	21/70 (30.0)
	Myalgia	18/163 (11.0)	5 /70 (7.1)
	Paraesthesia	1/163 (0.6)	1/70 (1.4)

	Radicular pain	1/163 (0.6)	0/70 (0)
Yang 2021 [12]	Headache	9/1607 (0.6)	1/770 (0.1)
	Myalgia	3/1607 0.2)	1/770 (0.1)
Chu 2021 [13]	Headache	301/796 (37.8)	69/393 (17.6)
	Myalgia	234/796 (29.4)	29/393 (7.4)
Walsh 2020 [14]	Headache	136/300 (45.3)	8/75 (10.7)
	Myalgia	86/300 (28.7)	4/75 (5.3)
Mulligan 2020 [15]	Headache	42/60 (70.0)	3/15 (20.0)
	Myalgia	23/60 (38.3)	0/15 (0)
		2027/6933 (29.2) *	570/2641 (21.6) *

* compared with control, P<0.05. Except for annotation, the number were person-time. Injection of the same person experiencing twice would be recorded as two.

Table S2. The incidences of headache and myalgia with or without prophylactic paracetamol [7]

Group		No. of events (%)	
		**Headache**	**Myalgia**
Paracetamol	Vaccine (n=56)	34 (60.7) *	27 (48.2) *
	Control (n=57)	21 (36.8)	15 (26.3)
No paracetamol	Vaccine (n=487)	331 (68.0) *	294 (60.4) *
	Control (n=477)	195 (40.9)	118 (24.7)

* compared with placebo, P＜0.05.

Table S3. Nervous and muscular adverse events in phase 1/2 open-label clinical trial on COVID-19 vaccination

Study	Vaccine	Group	No. of events (%)		
			**Headache**	**Myalgia**	**Other NMAEs**
Logunov 2020 [16]	replication-incompetent vectors vaccine	Single rAd26 or rAd5 (n=36)	16 (44.4)	8 (22.2)	NA
		rAd26 and rAd5 (n=40)	16 (40.0)	11 (27.5)	NA
Zhu 2020(2) [17]	replication-incompetent vectors vaccine	$5x10^{10}$ viral particles/mL (n=36)	14 (38.9)	7 (19.4)	1 (2.8) [b]
		$1x10^{11}$ viral particles/mL (n=36)	11 (30.6)	3 (8.3)	0 (0)
		$5x10^{11}$viral particles/mL (n=36)	17 (47.2)	8 (22.2)	1 (2.8) [b]
Jackson 2020 [18] and Anderson 2020 [19]	mRNA	1st Vaccination (n=85)	19 (22.4)	14 (17.1)	0 (0)
		2nd Vaccination (n=82)	43 (50.6) [a]	45 (54.9) [a]	2 (2.4) [c]
Total			136/351 (38.7)	96/351 (27.4)	4/275 (1.5)

* compared with the 1st vaccination, P＜0.05. † dizziness. ‡ syncope and lightheadedness. NMAEs= nervous and muscular adverse events. NA= not available.

Table S4. Headache and myalgia in phase 3 clinical trial on COVID-19 vaccination*

	Inactivated vaccine			Replication-incompetent vectors vaccine				mRNA vaccine				Total	
	Kaabi 2021 [20]†			Voysey 2020 [21]		Logunov 2021 [22]‡		Baden 2020 [23] §					
	Vaccine		Control (alum adjuvant) n=13453	Vaccine ($5x10^{10}$ viral particles/mL) n=12021	Control (MenACWY vaccine) n=11724	Vaccine n=1029	Control (vaccine buffer) n=340	Vaccine		Control			
	WIV04 n=13464	HB02 n=13471						1st injection n=15168	2nd injection n=14677	1st injection n=15155	2nd injection n=14566	Vaccine	Control
**Headache No. (%)**	1733 (12.9)	1761 (13.1)	1673 (12.4)	NA	NA	30 (2.9)	9 (2.6)	4951 (32.6) ¶	8602 (58.6) ¶, ‖	4027 (26.6)	3410 (23.4)	17077/57809 (29.5) ¶	9139/43514 (21.0)
**Myalgia No. (%)**	722 (5.4)	739 (5.5)	730 (5.4)	1 (<0.1)	0 (0)	9 (0.9)	3 (0.9)	3441 (22.7) ¶	8508 (58.0) ¶, ‖	2071 (13.7)	1809 (12.4)	13420/69830 (19.2) ¶	4613/55238 (8.4)

* Studies contained data of phase 3, and partly contained phase 1 and/or 2. † Observation was seven days. And a participant was only counted once in the specific reaction category even though a participant could have more than 1 adverse reaction. ‡ Adverse events registered during the analyzed period and the subjects were over 60 years old in the vaccine/control group. § Solicited NMAEs within 7 days after vaccination in safety set were counted. ¶ Compared with control, $P<0.05$. ‖ Compared with the 1st injection, $P<0.05$. NA= not available.

Table S5. Headache and myalgia subgroup in study of Baden 2020 [23] *

	mRNA vaccine							
	18~65 years old				⩾65 years old			
	Vaccine		Control		Vaccine		Control	
	1st injection n=11406	2nd injection n=10985	1st injection n=11407	2nd injection n=10918	1st injection n=3762	2nd injection n=3692	1st injection n=3748	2nd injection n=3648
**Headache No. (%)**	4030 (35.3) [†,§]	6898 (62.8)[†,‡,§]	3304 (29.0)	2760 (25.3)	921 (24.5)	1704 (46.2) [†,‡]	723 (19.3)	650 (17.8)
**Myalgia No. (%)**	2699 (23.7) [†,§]	6769 (61.6) [†,‡,§]	1628 (14.3)	1411 (12.9)	742 (19.7) [†]	1739 (47.1) [†,‡]	443 (11.8)	398 (10.9)

* Solicited NMAEs within 7 days after vaccination in safety set were counted. † Compared with corresponding control, $P < 0.05$. ‡ Compared with 1st vaccination of the same group, $P < 0.05$. § Compared with older (⩾65 years old), $P < 0.05$. No.= number.

References

1. Xia S.; Zhang Y.; Wang Y.; et al. Wang H.; Yang Y.; Gao G.F.; Tan W.; Wu G.; Xu M.; Lou Z.; et al. Safety and immunogenicity of an inactivated SARS-CoV-2 vaccine, BBIBP-CorV: A randomised, double-blind, placebo-controlled, phase 1/2 trial. *Lancet Infect. Dis.* **2021**, *21*, 39–51.
2. Xia S.; Duan K.; Zhang Y.; Zhao D.; Zhang H.; Xie Z.; Li X.; Peng C.; Zhang Y.; Zhang W.; et al. Effect of an inactivated vaccine against SARS-CoV-2 on safety and immunogenicity outcomes: Interim analysis of 2 randomized clinical trials. *JAMA* **2020**, *324*, 951–960.
3. Wu Z.; Hu Y.; Xu M.; et al. Chen Z.; Yang Wi;, Jiang Z.; Li M.; Jin H.; Cui G.; Chen P.; et al. Safety, tolerability, and immunogenicity of an inactivated SARS-CoV-2 vaccine (CoronaVac) in healthy adults aged 60 years and older: A randomised, double-blind, placebo-controlled, phase 1/2 clinical trial. *Lancet Infect. Dis.* **2021**, *21*, 803–812.
4. Zhang Y.; Zeng G.; Pan H.; Li C.; Hu Y.; Chu K.; Han W.; Chen Z.; Tang R.; Yin W.; et al. Safety, tolerability, and immunogenicity of an inactivated SARS-CoV-2 vaccine in healthy adults aged 18–59 years: A randomised, double-blind, placebo-controlled, phase 1/2 clinical trial. *Lancet Infect. Dis.* **2021**, *21*, 181–192.
5. Raches E.; Mohan K.V.; Harsh J.; et al. Sai P.; Siddharth R.; Vamshi S.; Brunda G.; Gajanan S.; Pragya Y.; Priya A.; et al.(2021). Safety and immunogenicity of an inactivated SARS-CoV-2 vaccine, BBV152: A double-blind, randomised, phase 1 trial. *Lancet Infect. Dis.* **2021**, *21*, 637–646.
6. Raches E.; Siddharth R.; Harsh J.; Vamshi S.; Brunda G.; Sai P.; Dipankar D.; Dugyala R.; Usha P.; Gajanan S.; et al. Safety and immunogenicity of an inactivated SARS-CoV-2 vaccine, BBV152: Interim results from a double-blind, randomised, multicentre, phase 2 trial, and 3-month follow-up of a double-blind, randomised phase 1 trial. *Lancet Infect. Dis*. **2021**, *21*, 950–961.
7. Folegatti P.M.; Ewer K.J.; Aley P.K.; Angus B.; Becker S.; Belij-Rammerstorfer S.; Bellamy D.; Bibi S.; Bittaye M.; Clutterbuck E.A.; et al. Safety and immunogenicity of the ChAdOx1 nCoV-19 vaccine against SARS-CoV-2: A preliminary report of a phase 1/2, single-blind, randomised controlled trial. *Lancet* **2020**, *396*, 467–478.
8. Zhu F.; Guan X.; Li Y.; Huang J.; Jiang T.; Hou L.; Li J.; Yang B.; Wang L.; Wang

We.; et al. Immunogenicity and safety of a recombinant adenovirus type-5-vectored COVID-19 vaccine in healthy adults aged 18 years or older: A randomised, double-blind, placebo-controlled, phase 2 trial. *Lancet* **2020**, *396*, 479–488.

9. Keech C.; Albert G.; Cho I.; Robertson A.; Reed P.; Neal S.; Plested J.S.; Zhu M.; Cloney-Clark S.; Zhou H.; et al. Phase 1–2 trial of a SARS-CoV-2 recombinant spike protein nanoparticle vaccine. *New Engl. J. Med.* **2020**, *383*, 2320–2332.
10. Richmond P.; Hatchuel L.; Dong M.; Ma B.; Hu B.; Smolenov I.; Li P.; Liang P.; Han H.; Liang J., et al. Safety and immunogenicity of S-Trimer (SCB-2019), a protein subunit vaccine candidate for COVID-19 in healthy adults: A phase 1, randomised, double-blind, placebo-controlled trial. *Lancet* **2021**,397, 682–694.
11. Chappell K.J.; Mordant F.L.; Li Z.; Wijesundara D.K.; Ellenberg P.; Lackenby J.A.; Cheung S.T.M.; Modhiran N.; Avumegah M.S.; Henderson C.L.; et al. Safety and immunogenicity of an MF59-adjuvanted spike glycoprotein-clamp vaccine for SARS-CoV-2: A randomised, double-blind, placebo-controlled, phase 1 trial. *Lancet Infect. Dis.* **2021**; doi:10.1016/S1473-3099(21)00200-0.
12. Yang S.; Li Y.; Dai L.; et al. Wang J.; He P.; Li C.; Fang X.; Wang C.; Zhao X.; Huang E.; et al. Safety and immunogenicity of a recombinant tandem-repeat dimeric RBD-based protein subunit vaccine (ZF2001) against COVID-19 in adults: Two randomised, double-blind, placebo-controlled, phase 1 and 2 trials. *Lancet Infect. Dis.* **2021**, *21*, 1107–1119.
13. Chu L.; McPhee R.; Huang W.; Bennett H.; Pajon R.; Nestorova B.; Leav B.; mRNA-1273 Study Group. A preliminary report of a randomized controlled phase 2 trial of the safety and immunogenicity of mRNA-1273 SARS-CoV-2 vaccine. *Vaccine* **2021**, *39*, 2791–2799.
14. Walsh E.E.; Frenck R.W.; Falsey A.R.; Kitchin N.; Absalon J.; Gurtman A.; Lockhart S.; Neuzil K.; Mulligan M.J.; Bailey R.; et al. Safety and immunogenicity of two RNA-based Covid-19 vaccine candidates. *New Engl. J. Med.* **2020**, *383*, 2439–2450.
15. Mulligan M.J.; Lyke K.E.; Kitchin N.; Absalon J.; Gurtman A.; Lockhart S.; Neuzil K.; Raabe V.; Bailey R.; Swanson K.A.; et al. Phase I/II study of COVID-19 RNA vaccine BNT162b1 in adults. *Nature* **2020**, *586*, 589–593.
16. Logunov D.Y.; Dolzhikova I.V.; Zubkova O.V.; Tukhvatulin A.I.; Shcheblyakov D.V.; Dzharullaeva A.S.; Grousova D.M.; Erokhova A.S.; Kovyrshina A.V.;

Botikov A.G.; et al. Safety and immunogenicity of an rAd26 and rAd5 vector-based heterologous prime-boost COVID-19 vaccine in two formulations: Two open, non-randomised phase 1/2 studies from Russia. *Lancet* **2020**, *396*, 887–897.

17. Zhu F.; Li Y.; Guan X.; Hou L.; Wang W.; Li J.; Wu S.; Wang B.; Wang Z.; Wang L.; et al. Safety, tolerability, and immunogenicity of a recombinant adenovirus type-5 vectored COVID-19 vaccine: A dose-escalation, open-label, non-randomised, first-in-human trial. *Lancet* **2020**, *395*, 1845–1854.
18. Jackson L.A.; Anderson E.J.; Rouphael N.G.; Roberts P.C.; Makhene M.; Coler R.N.; McCullough M.P.; Chappell J.D.; Denison M.R.;, Stevens L.J.; et al. An mRNA vaccine against SARS-CoV-2 - preliminary report. *New Engl. J. Med.* **2020**, *383*, 1920–1931.
19. Anderson E.J.; Rouphael N.G.; Widge A.T.; Jackson L.A.; Roberts P.C.; Makhene M.; Chappell J.D.; Denison M.R.; Stevens L.J.; Pruijssers A.J.; et al. Safety and immunogenicity of SARS-CoV-2 mRNA-1273 vaccine in older adults. *New Engl. J. Med.* **2020**, *383*, 2427–2438.
20. Kaabi A.I.N.; Zhang Y.; Xia S.; Yang Y.; Al Qahtani M.M.; Abdulrazzaq N.; Al Nusair M.; Hassany M.; Jawad J.S.; Abdalla J.; et al. Effect of 2 inactivated SARS-CoV-2 vaccines on symptomatic COVID-19 infection in adults: A randomized clinical trial. *JAMA* **2021**, *326*, 35–45.
21. Voysey M.; Clemens S.A.C.; Madhi S.A.; Weckx L.Y.; Folegatti P.M.; Aley P.K.; Angus B.; Baillie V.L.; Barnabas S.L.; Bhorat Q.E.; et al. Safety and efficacy of the ChAdOx1 nCoV-19 vaccine (AZD1222) against SARS-CoV-2: An interim analysis of four randomised controlled trials in Brazil, South Africa, and the UK. *Lancet* **2021**, *397*, 99–111.
22. Logunov D.Y.; Dolzhikova I.V.; Shcheblyakov D.V.; Tukhvatulin A.I.; Zubkova O.V.; Dzharullaeva A.S.; Kovyrshina A.V.; Lubenets N.L.; Grousova D.M.; Erokhova A.S.; et al. Safety and efficacy of an rAd26 and rAd5 vector-based heterologous prime-boost COVID-19 vaccine: An interim analysis of a randomised controlled phase 3 trial in Russia. *Lancet* **2021**, *397*, 671–681.
23. Baden L.R.; Sahly H.M.; Essink B.; Kotloff K.; Frey S.; Novak R.; Diemert D.; Spector S.A.; Rouphael N.; Creech C.B.; et al. Efficacy and safety of the mRNA-1273 SARS-CoV-2 vaccine. *New Engl. J. Med.* **2021**, *384*, 403–416.
